# Multiple endosymbionts in populations of the ant *Formica cinerea*

**DOI:** 10.1186/1471-2148-10-335

**Published:** 2010-11-01

**Authors:** Anu Sirviö, Pekka Pamilo

**Affiliations:** 1 Department of Biology, P.O. Box 3000, University of Oulu 90014, Oulu, Finland; 2 Department of Biological and Environmental Sciences, University of Helsinki 00014, P.O. Box 65, Helsinki, Finland

## Abstract

**Background:**

Many insects, including ants, are infected by maternally inherited *Wolbachia *endosymbiotic bacteria though other secondary endosymbionts have not been reported in ants. It has been suggested that the ability of *Wolbachia *to invade and remain in an ant population depends on the number of coexisting queens in a colony. We study the genetic and social structure of populations in the ant *Formica cinerea *which is known to have populations with either monogynous or polygynous colonies. We screen populations for several endosymbiotic bacteria to evaluate the presence of different endosymbionts, possible association between their prevalence and the social structure, and the association between endosymbiont prevalence and genetic differentiation of ant populations.

**Results:**

We found three endosymbiotic bacteria; 19% of the nests were infected by *Wolbachia*, 3.8% by *Cardinium *and 33% by *Serratia*. There was significant variation among the populations regarding the proportion of nests infected by *Serratia*, *Wolbachia *and the pooled set of all the endosymbionts. Some individuals and colonies carried two of the bacteria, the frequency of double infections agreeing with the random expectation. The proportion of infected ants (individuals or colonies) did not correlate significantly with the population level relatedness values. The difference in the prevalence of *Wolbachia *between population pairs correlated significantly with the genetic distance (microsatellites) of the populations.

**Conclusions:**

The discovery of several endosymbionts and co-infections by *Wolbachia *and *Cardinium *demonstrate the importance of screening several endosymbionts when evaluating their possible effects on social life and queen-worker conflicts over sex allocation. The low prevalence of *Wolbachia *in *F. cinerea *departs from the pattern observed in many other *Formica *ants in which all workers have been infected. It is likely that the strain of *Wolbachia *in *F. cinerea *differs from those in other *Formica *species. The correlation between the difference in *Wolbachia *prevalence and the pair-wise genetic distance of populations suggests that spreading of the bacteria is restricted by the isolation of the host populations.

## Background

Insects carry several endosymbiotic bacteria which can affect the life of their hosts [1]. Primary (or obligate) endosymbionts are usually mutualistic, host-specific and are restricted to vertical transmission in the maternal line and have long-term coevolution with their host. These include *Buchnera *in aphids, *Blochmannia *in *Camponotus *carpenter ants, *Wigglesworthia glossinidium *in tsetse flies and *Baumannia cicadellinicola *in leafhoppers [2]. Secondary (or facultative) endosymbionts can be either parasitic or mutualistic, they are found in a variety of host taxa, and are characterised by obvious horizontal transmission. Screening of secondary endosymbionts in insects is often restricted to *Wolbachia *which is inherited vertically from mother to offspring and can manipulate the reproduction of the host in ways that increase the number of female offspring [3]. Other secondary endosymbionts, including *Rickettsia*, *Spiroplasma *and *Cardinium *have been found in various groups of arthropods and they are also characterized by vertical transmission and reproductive manipulation [1]. They have been shown to induce cytoplasmic incompatibility, parthenogenesis, feminisation of male embryos and male killing [2,4]. In addition to the manipulation of reproduction, some of the secondary endosymbionts can give direct benefits to the host by delivering nutrients, by giving defence against natural enemies, by improving thermal tolerance, by enhancing fecundity and by inducing variation in breeding behaviour of the host [2,5]. Such features have raised questions about the role of endosymbiotic bacteria in inducing and controlling various phenotypic features of their arthropod hosts.

Reproductive manipulation by endosymbionts can be particularly important in the evolution of social Hymenoptera (ants, social bees and wasps) as it interferes with the sex ratio conflict between queens and workers [3,6,7]. Queens have commonly equal interest in the production of both sexes but workers can manipulate the sex ratio by preferentially caring either male or female larvae according to the relatedness asymmetry. If relatedness between nest mates is high workers favor the survival of females and when it is low they may care more male offspring. Workers of many social insect species do not reproduce, and if they do, they produce haploid males and thus prevent vertical transmission of endosymbionts. Yet, workers are numerous and form an important source for horizontal transmission. Mutualistic endosymbionts can also enhance the evolution of social behaviour and group living by forcing individuals to interact in order to obtain or receive benefits delivered by beneficial bacteria [see [8]].

Cytoplasmic incompatibility appears to be the most widespread *Wolbachia*-induced manipulation in insects and causes a serious load on the host population when the prevalence is close to 50% and incompatible matings are common. Selection is therefore expected to drive the prevalence towards zero or 100% [9]. In social insects colonies with many reproductive queens (polygynous colonies), the cost of incompatible mating can be reduced if other queens in the colony have compatible mating and produce a healthy colony. Based on this argument, it has been suggested that ants with polygynous colonies may have an intermediate prevalence of *Wolbacia *infection, whereas ants with monogynous colonies suffer from incompatible mating and should either lack infection or be all infected [10,11]. It should, however, be pointed out that the influence of *Wolbachia *on ant hosts has not been confirmed yet. Few laboratory and molecular studies have been done, without clear results despite widespread occurrence of different *Wolbachia *strains in various ant species [12]. So far, the studies have largely focused on the prevalence and patterns of transmission [13-16]. There are very few studies that have examined infection of social insects by bacteria other than *Wolbachia *[3,17,18] or the overall diversity of microorganisms in ants [19,20].

Because of the potential importance of endosymbiotic bacteria in social insect biology, we aimed to study the prevalence of several host-manipulating endosymbionts in the ant *Formica cinerea*, known also as the velvet or silky ant. The species inhabits open sandy areas and is distributed from South Western Asia to Central Europe, with local and seemingly isolated populations in northern Europe. Even though isolated on the map, the North-European populations have been found to be genetically relatively homogeneous with no obvious long-term isolation [21]. *Formica cinerea *is on a short list of ant species in which the social organisation of colonies varies among populations in such a way that colonies in some populations normally have a single queen (monogynous type) while they have multiple queens (polygynous type) in other populations [21,22]. This allows testing the hypotheses relating the prevalence of endosymbionts to the colonial type. Our study included ten populations in one geographical area (at most 50 km distance between populations) and we characterised the social type of colonies and inferred the gene flow between populations by using genetic markers (nuclear and mitochondrial), and screened the occurrence of several endosymbionts. Major questions we aimed to answer were: 1) Are there multiple endosymbionts in *F. cinerea *and do the endosymbionts compete with each other or do multiple infections occur frequently? 2) Is the prevalence of endosymbionts associated with the social type of colonies and is there any association between the prevalence of endosymbionts and the genetic distance between pairs of populations? Furthermore, we tested whether the social type influences gene flow in the ants as observed in other socially polymorphic ants [23]. We found three endosymbiotic bacteria (*Candidatus Cardinium*, *Candidatus Serratia symbiotica *and *Wolbachia*) infecting *F. cinerea*. This is the first study reporting potentially manipulative endosymbionts other than *Wolbachia *in ants.

## Results

### Genetic structure of the ant populations

The total number of alleles per microsatellite locus varied between 8 (FL20) and 12 (Fe16) across all the populations, and single populations had from 3 to 9 alleles per locus. The overall inbreeding coefficient was significantly positive in population 5 (Koppana sand pit, F = 0.16 with 95% CI 0.06 - 0.27) (Table 1). The statistical tests are problematic because related individuals are not independent of each other, but there was no clear tendency for any specific locus to show departures from the expected Hardy-Weinberg frequencies.

**Table 1:** Sample size, genetic characteristics of the ant populations and the number of nests affected by the endosymbionts.

Population		N_n_	N_all_	H_exp_	r		F	Number of infected nests					
							
					mean	se		*Serr*	*Wolb*	*Card*	*SW*	*SC*	*WC*
1	Juurussuo	22	28	0.74	0.54	0.06	0.02	7	2	2	0	1	1
2	Koppana beach	20	23	0.73	0.12	0.03	-0.01	7	6	1	3	1	0
3	Haukipudas	20	24	0.75	0.49	0.07	0.00	2	5	0	1	0	0
4	Jääli	17	26	0.73	0.45	0.05	0.04	4	2	0	1	0	0
5	Koppana sand pit	16	27	0.75	0.40	0.08	0.16	13	1	1	0	1	0
6	Liminka	9	17	0.67	0.59	0.05	-0.16	0	2	0	0	0	0
7	Mäntyniemi (Hailuoto)	9	26	0.55	0.44	0.09	-0.01	5	6	0	4	0	0
8	Tauvo	12	23	0.70	0.04	0.04	0.01	3	0	0	0	0	0
9	Keskiniemi (Hailuoto)	3	16	0.60	-	-	-	1	0	0	0	0	0
10	Huilunnokka (Hailuoto)	4	17	0.71	-	-	-	1	1	1	0	0	1

N_n _and N_all _refer to the number of nests and microsatellite alleles. Five worker ants were screened from each nest. H_exp _= expected heterozygosity, r = relatedness and F = inbreeding coefficient. The standard error of relatedness was obtained by jackknifing over the nests. Number of infected nests is given for each bacterium (*Serr, Wolb, Card*) and for double infections (SW, SC and WC).

Mean genetic relatedness (r) of colonial nest-mates within populations varied from 0.04 to 0.59 (Table 1) indicating different social forms in the populations. The relatedness estimates divided the populations in two groups (Table 1). The relatedness estimates in Tauvo (r = 0.04) and Koppana beach (r = 0.12) were significantly smaller than in the other six populations (in which r ≥ 0.40), whereas there were no significant relatedness differences among the populations within the two groups (pair-wise comparisons with *t*-test). The level of polygyny in the Koppana sand pit population was unclear as the estimate of relatedness was r = 0.40 but there was also an excess of homozygotes, and the inbreeding-adjusted relatedness [24] was r* = 0.17. The high initial relatedness, however, indicates that the nests were genetically separate and did not form colonial networks exchanging workers.

Colonies within populations had a wide range of relatedness values, but it should be noted that such individual estimates have a large sampling variance. The two populations with low average worker relatedness had 10% of nests (3 out 32) with relatedness r > 0.3. Likewise, the six populations with a high average relatedness had 10% of nests (8 out of 82) with relatedness r < 0.11.

The pair-wise estimates of F_ST _ranged from 0.01 to 0.17 (Table 2). Populations 1, 3, 4 and 5 did not differ significantly from each other (permutation test, P > 0.05), but the other populations showed significant differences (F_ST _larger than obtained by permutation of the nests) to any other population. The population on the island of Hailuoto (7, Mäntyniemi) was the most differentiated population (Table 2, Additional File 1). The correlation between the genetic distance and the geographical distance was not significant (r_m _= 0.20, P = 0.25, Mantel's test). Notably, the two closely located Koppana populations (2 and 5) that were separated by only 1.5 km differed significantly from each other with F_ST _= 0.05.

**Table 2:** Pair-wise genetic differentiation (F_ST_) of the ant populations.

	1	2	3	4	5	6	7
2	0.033						
3	0.011	0.020					
4	0.013	0.056	0.009				
5	0.010	0.036	0.008	0.005			
6	0.041	0.108	0.051	0.091	0.056		
7	0.110	0.154	0.084	0.059	0.068	0.149	
8	0.009	0.023	0.018	0.066	0.033	0.045	0.169

The mitochondrial sequences revealed five haplotypes A-E (Additional file 2) [GenBank: haplotype A: GU592755, B: GU592752, C: GU592754, D: GU592751 and E: GU592753]. Haplotype E was prevailing and included most samples from all the populations. Haplotype A was found in four individuals from two colonies of population 4 (Jääli) and had a single transition in the tRNA-Ser part of the amplified region. Haplotypes B and C had single nucleotide changes, both leading to an amino acid change (I to V and L to I). Haplotype B was detected in individuals from four colonies of population 5 (Koppana sand pit). Haplotype C occurred in two populations, in individuals from four colonies of population 1 (Juurussuo) and from three colonies of population 4 (Jääli). Haplotype D could be derived from the haplotype C by a synonymous transition and was observed in only a single colony in population 3 (Haukipudas). In the populations that had two haplotypes, only one haplotype was found in each nest.

#### *Wolbachia*

No amplification was initially detected when the *wsp*-primer pair was used for all 656 *F. cinerea *individuals, while positive controls (*F. aquilonia*, *F. rufa, F. polyctena*, *F. lugubris*, *F. paralugubris, F. lemani*) produced a strong band that was confirmed to belong to *Wolbachia *by sequencing. Different concentrations (20-60 ng/ul) of genomic template were tested for a subset of samples, as well as different annealing temperatures (50-60°C) and several MgCl_2 _concentrations (1-3 mM) but PCR produced either almost invisibly faint bands that had a correct fragment size or mostly no bands at all for *F. cinerea wsp*. When hemi-nested PCR with several bacteria-specific primer pairs was used to amplify 16SrDNA, we successfully amplified *Wolbachia, Serratia *and *Cardinium *from *F. cinerea*. The other bacteria (*Ricketsia*, *Spiroplasma*, *Buchnera*) were not detected.

**Figure 1:**
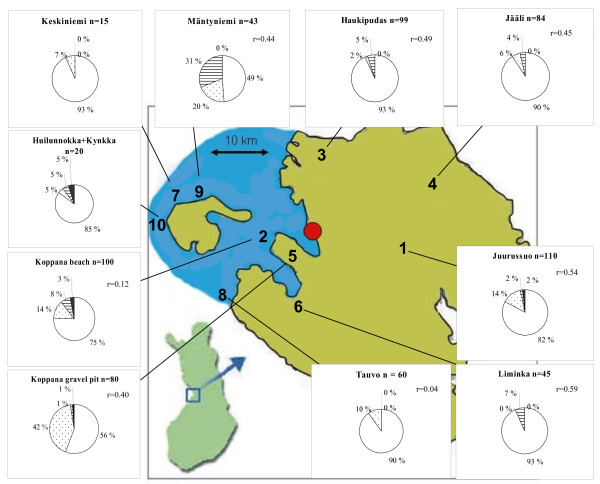
**Infection percentages and the relatedness values in *F. cinerea *populations**. The percentage of individuals infected by different endosymbionts are shown as segments in pie diagrams where the dotted areas represent *Candidatus Serratia symbiotica*, horizontal lines *Wolbachia*, black areas *Cardinium *and the white areas uninfected individuals. The relatedness values and the total number of individuals are shown for each population. The segments of the diagrams do not properly take into account the few individuals that were multiply infected. The scale of the map is proportional.

*Wolbachia *was found in eight out of ten populations and the proportion of infected individuals in those ranged from 1.2% (population 5) to 33% (population 7) (Table 1, Figure 1). In total, 5.6% of the individuals and 19% of the nests were infected by *Wolbachia. *Some singleton sites of the obtained sequence (896 bp) can represent PCR errors, but there were 49 parsimony informative sites. Among the haplotypes, four from Liminka (population 6) differed from all the others at 21-27 sites (mean difference p = 0.025). Among the other haplotypes, the largest number of pair-wise nucleotide differences was 13 (p = 0.014) and the largest differences were between sequences from Haukipudas (population 3) and the island of Hailuoto (populations 7, 10). In a neighbour-joining tree, the sequences from these two areas tended to form groups of their own, otherwise there were no clear geographical groupings (Additional file 3). A BLAST search showed that the majority of *Wolbachia *sequences of the 16SrDNA from *F. cinerea *[GenBank GU592769-GU592781] were 98-99% similar to *Wolbachia*-like uncultured bacterial clones from the ant lion *Myrmeleon mobilis*, fly *Cacoxenus indagator*, and giant scale insect *Drosicha pinicola *as well as to *Wolbachia *from several *Drosophila *species and *Diabroticite *beetles (Additional files 3, 4 and 5).

In order to compare the *Wolbachia *in *F. cinerea *with the strains found earlier in other *Formica *ants, we sequenced bacteria from several *Formica *species (*F. aquilonia*: Finland, *F. polyctena*: Russia and Sweden, *F. lugubris*: France, *F. rufa*: Russia) that were known to carry different *wFex*-strains of *Wolbachia *determined by the *wsp *sequences [[15] and our unpublished sequences for *F. aquilonia*]. The 16SrDNA sequences obtained from these hosts were either identical to one of the sequences found in the major group from *F. cinerea *or differed by only one nucleotide (see Additional file 5). [GenBank:*F. rufa *GU592782, *F. aquilonia *GU592783-GU592785, *F. lugubris *GU592786, *F. polyctena *GU592787-GU592788, *F. lugubris *GU592789]. The deviating sequences from the Liminka population of *F. cinerea *fell outside this group.

#### *Cardinium*

*Cardinium *was present in seven individuals that came from four populations (1, 2, 5 and 10), and the proportion of infected individuals ranged from 1.3% (population 5) to 5% (population 10) (Table 1, Figure 1). In total, only 1.1% of the individuals and 3.8% of the nests were infected. The sequences (450 bp) had 11 parsimony informative sites [GenBank GU592742-GU592750]. The neighbour-joining tree of the haplotypes showed no clear geographical pattern.

**Figure 2:**
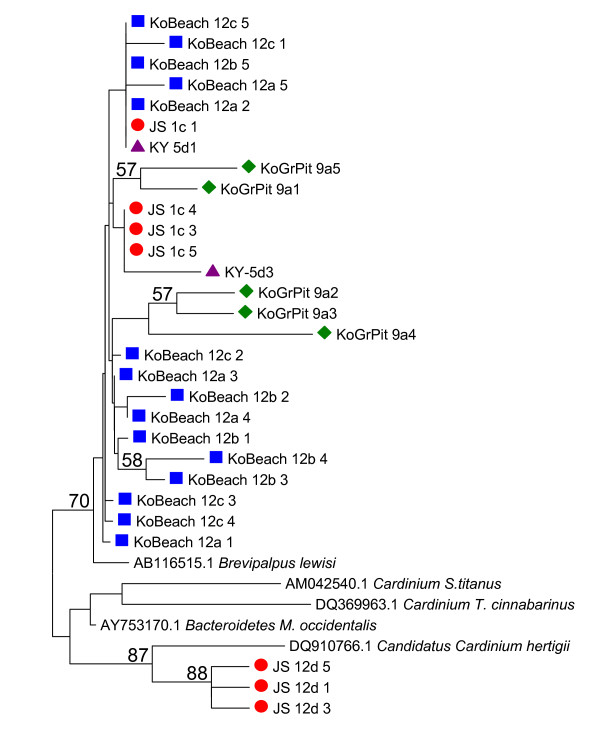
**The neighbour-joining tree of *Cardinium *sequences (450 bp)**. The tree has sequences amplified from *F. cinerea *in this study and some sequences derived from GenBank. The tree is based on distances estimated by using the Kimura's 2 parameter method with gaps and missing data excluded from pair-wise comparisons. JS: population 1, KoBeach: population 2, KoGrPit: population 5, and KY: population 10. (1000 bootstrap replicates).

The maximum number of pair-wise nucleotide differences was ten (p = 0.022, total length 450 bp). The parsimony informative sites separated three haplotypes in the Juurussuo population (population 1) from the others by five specific differences. The main group of sequences was 98-99% similar to *Cardinium *sequences obtained earlier from several species of *Brevipalpus *mites, *Metaseiulus occidentalis*, leafhopper *Scaphoideus titanus *and spider mite *Oligonychus ilicis *(Figure 2, Additional file 6).

#### *Serratia*

Sequences produced by the PASS primers [GenBank GU592756-GU592768] gave 98% similarities with symbiont S from *Acyrthosiphon *pea aphid (M27040), secondary symbiont type-R of *Aphis craccivora *(AY822593, AY822592, AY822591, AY822594), secondary symbiont of *Acyrthosiphon pisum *(AB033777, AB033778), and *Candidatus Serratia symbiotica *(AF293617, AY296732) (see Additonal file 7). Thus our sequences could be considered to belong to the recently named new species *Candidatus Serratia symbiotica *(formerly known as PASS or R-type secondary symbiont of aphids) [25]. *Serratia *was found in all populations except Liminka (population 6). Not all the nests in the infected populations had *Serratia*, and the proportion of infected individuals within populations ranged from 2% (population 3, Haukipudas) to 43% (population 5, Koppana sand pit) (Table 1, Figure 1), and a total of 13.2% of all the *F. cinerea *individuals and 33% of the nests were infected.

The sequence (471 bp) had 19 parsimony informative sites. The largest pair-wise difference was 7 nucleotides (p = 0.014), and the neighbour-joining tree showed no clear geographical clustering of the haplotypes (Additional file 8).

### Connections with the genetic structure of the ant populations and multiple infections

There was significant variation among the populations regarding the fraction of nests infected by *Serratia *(χ^2 ^= 26.2, df = 7, P < 0.001), by *Wolbachia *(χ^2 ^= 21.7, df = 7, P < 0.01) and by the pooled set of all the endosymbionts (χ^2 ^= 21.0, df = 7, P < 0.01). The infection frequencies of the two most common endosymbionts, *Serratia *and *Wolbachia*, showed no significant correlation when the frequencies of infected nests in the eight populations were compared (Spearman's r_s _= -0.09, df = 6, ns).

We next tested whether the observed heterogeneity in the infection frequencies was correlated with the genetic structure of the ant populations. No significant correlation between the fraction of infected nests and the mean relatedness in the eight populations was observed (r_s _= -0.45 for *Serratia*, r_s _= 0.19 for *Wolbachia *and r_s _= -0.43 when pooling all the endosymbionts, df = 6, ns). As the power of the test at the level of populations may be restricted by the small number of populations, we also tested for a possible association by pooling populations. For this we used only populations with a somewhat similar average relatedness (0.40 < r < 0.59) and tested whether the relatedness estimates calculated for each nest were different in infected and non-infected nests. No significant difference was found when testing for *Serratia*, *Wolbachia *or all the endosymbionts (Mann-Whitney test).

The number of workers infected by *Serratia *in a nest showed significant positive correlation with the nest relatedness in population 7 (Mäntyniemi) (Spearman r_s _= 0.70, n = 9, P < 0.05). No significant correlation was found in other populations or for *Wolbachia *infections.

Infection by all three bacteria was not found in any individuals or colonies, though some double infections were found within both individuals and colonies. *Serratia*-*Wolbachia *infections were detected in some individuals from population 7 (Mäntyniemi, 2 individuals out of 43), population 3 (Haukipudas, 1/99) and population 4 (Jääli, 1/84). Double infection by *Serratia *and *Cardinium *was found only in population 1 (Juurussuo) where a single ant out of 110 carried both bacteria. *Wolbachia-Cardinium *double infection was found in population 2 (Koppana beach) where two ants from the same colony were infected out of 100 individuals.

When pooling all the material, the frequency of infected nests was 42/132 for *Serratia*, 25/132 for *Wolbachia *and 5/132 for *Cardinium*. The observed number of nests with two different bacteria matched the random expectation well based on these frequencies, 9 nests with *Serratia *and *Wolbachia*, 3 nests with *Serratia *and *Cardinium *and 2 nests with *Wolbachia *and *Cardinium*.

As the spreading of endosymbionts should be associated with the dispersal of the ant hosts, we next tested whether the level of genetic differentiation between population pairs (F_ST_) was correlated with the difference in the infection frequency between the same population pairs. The difference in the prevalence of *Wolbachia *increased with the genetic distance between populations (r_m _= 0.62, P = 0.05, Mantel's test), but no correlation was found for *Serratia *(r_m _= 0.11, P = 0.25). The correlation between the geographical distance (measured in km) and the difference in the infection frequency was not significant for either *Wolbachia *(r_m _= 0.27, P = 0.17) or *Serratia *(r_m _= - 0.22, P = 0.88). The latter correlation was even negative.

The only indication for a possible association between mitochondrial haplotype and endosymbiont infection was seen in mtDNA haplotype B. It was found in four colonies and three of these carried also *Serratia *while no other endosymbionts were detected in these colonies. In total seven colonies (from two populations) had the haplotype C, and *Wolbachia *was detected in two of these, *Cardinium *in one and *Serrata *in none. The two colonies with haplotype A and the only colony with haplotype D had none of the endosymbionts.

## Discussion

We found that the *F. cinerea *ants were infected by multiple endosymbiotic bacterial species *Wolbachia*, *Cardinium *and *Serratia*. The prevalence of both *Wolbachia *and *Serratia *varied significantly among the populations and colonies. This variation allowed the investigation of whether the prevalence of endosymbionts was associated with the social structure of colonies [10,11], whether the infections were associated with specific genetic lines defined by mitochondrial haplotypes [13] or genetic differentiation of populations, and whether the endosymbiont species competed with each other or co-infected receptive hosts. This is the first study to reveal *Cardinium *and *Serratia *in ants.

### Endosymbionts and the genetic structure of populations

Two of the detected endosymbionts, *Wolbachia *and *Cardinium*, are known as reproductive manipulators in insects. If the endosymbiotic bacteria cause cytoplasmic incompatibility in the host, the infection frequency is expected to be either low or high, as intermediate frequencies would commonly lead to incompatibilities [9]. The fraction of infected nests was low (3.8%) for *Cardinium *and moderate for *Wolbachia *(19.0%). Our results on *Wolbachia *depart from the general pattern observed in *Formica *ants, as in several species in northern Europe all or almost all individuals have been infected by the same *Wolbachia *strains [15,26]. The prevalence in *F. cinerea *was low and the *wsp *primers did not amplify the bacteria, indicating that *F. cinerea *could be infected by a different strain. The frequency of *Cardinium *infections was very low for reliable statistical comparisons, but the two endosymbionts seemed to coexist as expected on the basis of random association. In other studies, double infections of unrelated manipulative bacterial species have been reported to be relatively rare [1]. The density and location of endosymbionts within the host has been shown to depend on co-infection, as observed for *Wolbachia *and *Spiroplasma*, as well as on the host genotype and environmental conditions [27,28]. If different bacteria, or different strains of the same bacteria, cause reproductive incompatibility, selection could increase the frequency of all of them and lead to multiple infections as observed for *Wolbachia *in the ants *Formica exsecta *[26] and *Acromyrmex *[29].

Wenseleers et al. [10] proposed that while *Wolbachia *may have problems invading an ant population with monogynous colonies, it could spread in populations with polygynous colonies. Their data also supported this hypothesis as *Wolbachia *was found more commonly in polygynous species of South-East Asian ants than in monogynous species. Later, Shoemaker et al. [11] predicted that in monogynous species selection will tend to maintain high rates of infection because of the high costs of incompatible matings. This, indeed, seemed to be the case in *Solenopsis invicta *fire ants. Thus, the hypotheses make opposing predictions concerning the association between the infection frequency and the level of polygyny. The arguments were based on the fact that some strains of *Wolbachia *are known to cause cytoplasmic incompatibility in insects, but it should be noted that the effects of endosymbionts on ant reproduction have not been documented. *Formica cinerea *is known to show social polymorphism in the sense that colonies tend to be monogynous in some populations while polygynous in others [21,22]. The colonies in polygynous populations can also form large supercolonies that consist of networks of interconnected nests. Such differences in social organisation were also detected in the present study with genetic relatedness among worker nest mates ranging from 0.04 to 0.59. However, the fraction of infected *F. cinerea *ants (individuals or colonies) was not significantly correlated with the relatedness. We can conclude that no clear association existed between the endosymbionts and the social type of the colonies.

There are three possible explanations for why the prevalence of *Wolbachia *in *F. cinerea *was low compared to the other European *Formica *ants. First, our material consisted of worker ants and it has been suggested that workers can clear an infection, even though no clearance has been found in several *Formica *species that have multiple infections [15,26]. Infected workers may suffer an energetic cost which harmfully affects the whole colony, both ants and endosymbionts [7]. Some previous ant-*Wolbachia *studies have revealed that the infection level tends to decrease with development; the worker brood and female reproductives have higher infection levels than the adult workers and males [7,30]. Such clearance does not necessarily harm the interests of the bacteria as a non-reproductive worker can be a dead-end to a vertically transmitted endosymbiont. However, due to the large number of worker individuals, they offer a suitable source for horizontal transfer of endosymbionts and it is therefore not evident that clearance is beneficial for the bacteria. The infection frequencies in *F. cinerea *were so small that it is doubtful whether they could be explained by clearance, and it is reasonable to suggest that not all the nests had infected queens.

Second, the ants may have lost infections due to population processes. For example the *Wolbachia *prevalence in invasive ant species is lower in the introduced range than the native range [12,14,31]. The complete lack of *Wolbachia *or low level of infection has been considered as a sign of success of ants without the *Wolbachia *load in new habitats [11,31]. *Formica cinerea *has distributed to northern latitudes after the withdrawal of the last glacial period and is specialised in living in sandy habitats that often appear as patchy areas. The habitat patches (open sandy areas) are temporary and the populations have been forced to track them, creating local bottlenecks as is seen in invading species. In agreement with this view, genotypic variation indicates past contacts between the *F. cinerea *populations in northern Europe even though they appear isolated today [21]. The low overall level of nucleotide diversity in mtDNA found in this study is also compatible with this view and resembles the situation in several other *Formica *ants in northern Europe [32].

Third, the low prevalence in *F. cinerea *compared to other *Formica *species could result from an infection by a different strain of *Wolbachia*. The commonly used primers for the *wsp *gene failed to produce any visible band from *F. cinerea *samples even though the primers work well in other *Formica *ants [[10,15,26], this study]. We also sequenced 16SrDNA from several species from the *Formica rufa *group, known to harbour strains wFex1, wFex2 and wFex4 as defined by their *wsp *sequences [15]. The *wsp *sequences of these strains differ from each other by 10-21% of nucleotides. The 16SrDNA sequences from these samples were identical to or differed by only one nucleotide from the common sequences found in *F. cinerea*, and the most divergent 16SrDNA haplotypes observed from *F. cinerea *differed by about 3% from each other. 16SrDNA is more conservative than the *wsp *gene, and recombination between different *Wolbachia *strains may make it difficult to infer the type of strain from a single gene. Sequencing of *Wolbachia *genome from *Drosophila simulans *has revealed that *Wolbachia *wRi has the most highly recombining intracellular bacterial genome known to date [33]. Recombination in *Wolbachia *breaks the anticipated correlations between gene history, genome history and strain phenotype, thus no single sequence can correctly determine relationships between different *Wolbachia *strains [33]. Furthermore recent finding that massive amount of *Wolbachia *sequences (177 kb) have been transferred into the nuclear genomes of some hosts adds further complexity in interpretation of the infection status [33-35].

Some population pairs showed considerable genetic differentiation as indicated by the pair-wise F_ST _estimates and the genetic differentiation of the hosts was significantly correlated with a difference in *Wolbachia *prevalence. The two Koppana populations (beach and sand pit) give a good example of this as they showed a significant genetic difference in spite of their close proximity (separated by 1.5 km). The clustering of the mtDNA haplotype B in the Koppana sand pit population, even though in only four colonies, agreed with the pattern of differentiation seen in microsatellites. The Koppana populations showed widely different endosymbiont prevalences (Table 1, Figure 1). About 45% of individuals were infected in Koppana sand pit (pooling all the three bacteria) but only 25% in Koppana beach. The correlational evidence does not tell whether limited dispersal of the ants has restricted the spreading of the endosymbionts or whether incompatibilities caused by the endosymbionts have restricted the gene flow of the host. Intraspecific comparisons of nearby populations with different social organisation have in other *Formica *species shown that the polygynous colonies have less genetic variation than the neighbouring monogynous populations, particularly in mitochondrial DNA [*F. exsecta *[23] and *F. truncorum *[36]]. This has been taken to indicate that polygynous supercolonies may originate through a population bottleneck and grow by budding of colonies with restricted dispersal, as also observed in introduced ant species that form unicolonial populations [e.g. [37]]. In agreement with this, none of the rare mtDNA variants (A, B, C and D) in *F. cinerea *were found in the most polygynous populations.

### Endosymbiont diversity in *F. cinerea*

Various maternally inherited bacteria are estimated to infect approximately one third of arthropod species [1]. *Wolbachia *has been found to infect around 66% of the species whereas other reproductive endosymbionts (*Cardinium*, *Arsenophonus*, *Spiroplasma*) occur in 4% to 7% of studied arthropods [1,38]. At the population level, the range of *Wolbachia *infections has been reported from 3% to 100%, generally being fixed or close to fixation [1,39]. *Wolbachia *infections vary in *F. cinerea *between 1.2%-33% in populations and when taking into consideration the average infection percentage 5.6% we can conclude that *Wolbachia *has low prevalence in *F. cinerea*.

Six to seven percent of the studied insects and mites and 22% of spider species are reported to have *Cardinium *infection [40,41] and it has been reported from Hymenoptera, Hemiptera, Diptera, Acari and Aranea [1,42]. *Cardinium *has not been earlier reported from ants even though three ant species (*Cataglyphis *sp., *Messor capitatus*, *Platythyrea punctata*) were included along with 96 other invertebrates in a study where it was screened [40]. We found *Cardinium *at low frequency (1-2% of individuals) in *F. cinerea. Cardinium *is known to induce CI in parasitoid wasps *Encarsia *spp and in spider mites *Eotetranychus suginamensis *[43,44], parthenogenesis in parasitoid wasps *Encarsia *spp [45] and feminization in false spider mites *Brevipalpus *spp [2]. In predatory mite *Metaseiulus occidentalis*, *Cardinium *is reported to enhance the fecundity of the species [46]. Historical evidence for horizontal transfer has been reported for *Cardinium *[40], even though some studies failed to find evidence for it even between parasitoids and their hosts [e.g. [47]].

*Serratia symbiotica *is known as a secondary endosymbiont of aphids [25] and was detected in all but one population of *F. cinerea *with the infection percentage varying from 2% to 42%, and the overall percentage was 33% of the nests. In several populations *Serratia *was the most common of the endosymbionts studied here and in Koppana sand pit (population number 5) almost all the colonies (13/16) were infected. A few sequences of *Serratia *differed clearly from the others (by 1.4%), indicating two separate bacterial lineages. This difference is similar to that between *Serratia *haplotypes from *F. cinerea *and some aphids (0.4 - 1.5%). It is likely that the bacteria have been transferred horizontally both within and between species as has been detected in other *S. symbiotica *studies [25,48].

The fact that *Serratia *is found in both ants and aphids could be related to the fact that many ants, including *F. cinerea*, prey on aphids and also tend them for honeydew. This can offer a potential transmission route because honeydew does not contain only plant sap but also excretions from the aphid. Oral ingestion has been suggested to provide a transmission route also among aphids either via honeydew, squashed aphids or phloem sap of plants heavily populated by aphids [49]. It is not sure whether *F. cinerea *has *Serratia *as a true endosymbiont or whether it is only transient gut bacterium in ants. We successfully amplified both *Wolbachia *and *Cardinium *from the heads and thoraces of the ants, *Wolbachia *also from legs, suggesting that these bacteria exist as true endosymbionts. Other *Serratia *species (e.g. *S. marcescens*) are known to associate with many insects as a pathogen [50]. It will be interesting to explore the role of *Serratia *in the biology of *F. cinerea *as various gut symbionts have been shown to be a major force in ant evolution [20]. It has also been shown that *Wolbachia *can be an essential symbiont in insects, providing vitamins to the host [51]. Although many endosymbionts are mainly vertically transmitted, many cases of horizontal transfer between host species have been reported. Our study clearly indicates the need for a large scale screening not only for *Wolbachia *but also for other endosymbionts that are capable for manipulation of the reproduction and sexual bias of the host species.

## Conclusions

We found three endosymbiotic bacteria, *Wolbachia*, *Cardinium *and *Serratia *with significantly varying infection frequencies among *Formica cinerea *colonies and populations. This is the first report of *Cardinium *and *Serratia *in ants. Both *Wolbachia *and *Cardinium *are known to manipulate reproduction of their hosts, even though the effects have not been demonstrated in ants. Our discovery of several endosymbionts and co-infections by *Wolbachia *and *Cardinium *demonstrate the importance of screening several endosymbionts when evaluating their possible effects on social life and queen-worker conflicts over sex allocation. Whether *Serratia *is an endosymbiont or a transient gut bacterium in *F. cinerea *needs to be clarified. All three bacteria had low to moderate prevalence in ants. This differs from the pattern seen earlier in many *Formica *species in which all worker ants have been infected, and it is likely that *F. cinerea *carries a different strain of *Wolbachia*. The ant populations could be divided into two groups, one characterised by highly polygynous nests/relatedness among worker nest mates r ≤ 0.12) and the other by largely monogynous nests (r ≥ 0.40). The prevalence of the endosymbionts did not correlate significantly with the relatedness, suggesting that there is no evident association between the endosymbiont infections and the social type of the colonies at the geographical scale studied. The difference in *Wolbachia *prevalence correlated significantly with the pair-wise genetic distance of populations suggesting that spreading of the bacteria is connected to the isolation of the host populations. However, it is not possible to conclude whether limited dispersal of the ants has restricted the spreading of the endosymbionts or whether incompatibilities caused by the endosymbionts have restricted the gene flow of the host.

## Methods

### Samples

The material came from 656 worker ants sampled in 10 *Formica cinerea *populations (Table 1) around Oulu region in central Finland in September and October 2001. We sampled 3 to 22 colonies per population and five workers per colony. Only eight populations with at least nine nests were used for the population analyses. The distance between the sampled nests was at least 5 metres to increase the probability that different colonies were selected. The distance between populations varied from 1.5 to 50 km. Samples were stored in 90% ethanol (which helps to separate the ectoparasitic mites), and we also visually checked to make sure that the ants did not carry any mites. Genomic DNA was extracted using the Qiagen DNeasy Tissue Kit that purifies also mitochondrial and bacterial DNA. Individual ants were placed in separate 1.5 ml eppendorf tubes that were dipped in liquid nitrogen and ground by using disposable microtube pestles. The following steps in DNA extraction were done according to manufacturer's protocol for animal tissue. DNA concentration and purity of samples were measured by spectrophotometer (Eppendorf biophotometer) and concentration values ranged between 20-60 ng/ul (in final volume of 200 ul) and purity of DNA was measured as ratio of absorbances at wavelengths of 260 and 280 nm, the values ranged between 1.5-1.8 when using sterile water as diluent. We also checked the presence of *Wolbachia *and *Cardinium *in different body parts separately and succesfully amplified and sequenced them from DNA extracted from both heads and thoraces. *Wolbachia *was also found in gasters and legs of the ants.

### Mitochondrial sequences

Primer pair cytb3-tRNA-Ser [52] was used to amplify about 500 bp of mitochondrial DNA including the 3' region of the gene cytochrome oxidase b, short intervening region and 5' sequence of the gene tRNA-Ser. The total volume of the PCR reactions was 15 ul and contained 1× PCR buffer, 1.5 mM MgCl_2_, 0.25 mM of each nucleotide, 0.5 μM primers cytb and tRNASer, 0.3 units of enzyme (Dynazyme II by Finnzymes). The following PCR program was used for amplification: denaturation at 94°C for 3 min, 94°C for 1 min, 50°C 50 sec, 72°C for 1 min, cycles 30, final extension at 72°C for 10 min using PCR machine PTC-200. The PCR products were run in 2% agarose gels at 100 V for 2-3 hours to verify the success of amplification. Thereafter they were run on Single Strand Conformation Polymorphism (SSCP) gels to study haplotype variation. Three gel temperatures were tested, +4°C, +15°C and +37°C and temperature +37°C gave the best results and was used for all the samples. Prerun of SSCP was done at 200 V for 30 minutes to warm up the acrylamide gels and to remove additional traces of acrylamide from wells where denatured samples were run for 5 hours at 200 V. The gels were stained using the AgNO_3 _method [53] and dried on frames in room temperature for two days before scoring. One individual per nest and the samples that had a deviating banding pattern on SSCP were selected for further sequencing. To strengthen the intensity of the amplification products for direct sequencing, we did secondary PCR by using 1 ul of the initial PCR product as a template. Otherwise the protocol was the same as in the first PCR. Secondary PCR products were purified by using the MinElute kit (Qiagen) for direct sequencing reactions where 1/8 part reactions of Big Dye Terminator v 3.0 were done according to the instructions of the manufacturer (Applied Biosystems, Foster City, CA). Sequencing reactions were purified by the Sephadex-method (Sigma Aldrich) before the run in the capillary sequencer ABI3730. Sequences were inspected by the program Sequencher v 4.7 (Gene Codes Corporation) and alignments were done by ClustalW in MEGA v 3.1 [54].

### Microsatellites

Fifteen microsatellite loci were tested with gradient PCR (45-64°C) and the products were run in 2% agarose gel and in ABI377. Four highly polymorphic loci (Fe13, Fe16, Fe38, FL20) [55,56] were selected. The primer pairs Fe13, FL20 and FL20 had been used in an earlier study on *F. cinerea *[21,22]. The microsatellite PCR-program consisted of predenaturation at 94°C for 3 min, 35 cycles consisting 94°C for 1 min, 55°C for 40 sec (for Fe13 primer pair 53°C for 40 sec), 72°C for 50 sec. In the end an elongated extension time of 45 minutes was applied at 72°C to assure the proper extension of PCR products. PCR products were run in an ABI prism 377 Sequencer and fragments were analysed by using the GeneScan and Genotyper software packages (Applied Biosystems, Foster City, CA)

### Endosymbionts

The same DNA samples that were used in the analysis of nuclear and mitochondrial markers were studied for several endosymbionts. Thus we could be certain that the DNA extractions were successful and the genomic DNA was of good quality. The following primer pairs were tested for *Wolbachia *by direct PCR: *wsp *81F-691R [57], Wolb 16SrDNA [12] and general 16SrDNA [58]. PCR reactions for the *wsp*-primer pair contained 1× PCR buffer, 2 mM MgCl_2_, 0.25 mM of each nucleotide, 0.5 μM of primer 81F and 691R, and 0.3 U of DynazymeII (Finnzymes). Gradient PCR (50°C-60°C) was used to test optimal amplification conditions for *Wsp *by using *F. cinerea *individuals and positive controls (*F. aquilonia *and *F. lemani *individuals) as test samples. The annealing temperature that gave the strongest bands (for positive controls) was selected for use in a further PCR program for *wsp*. In addition, primer pairs were used for several additional positive controls from different *Formica *species, including *F. polyctena *(colonies U-11 and S-33 studied for mtDNA [32] and for *wsp *[15]), *F. rufa *(S-35), *F. lugubris *(S-42, U-36), *F. pratensis *(S-44). The PCR program included steps of denaturation at 94°C for 3 min, 94°C 1 min, 53°C 50 sec, 72°C 1 min, cycled 40 times, final extension at 72°C for 10 min. Positive (*F. lemani *and *F. aquilonia*) and negative (sterile water) controls were included in all the PCR-runs. The amount of template used was 1 ul (20 ng/ul).

Hemi-nested PCR [46] was used to screen several endosymbiotic bacteria. In the first PCR reactions we used universal primer pair 27Forward 5'-AGAGTTTGATCMTGGCTCAG-3' and 1513Reverse 5'-ACGGYTACCTTGTTACGACTT-3' for 16SrDNA that amplify a 1.5 kb fragment from all eubacteria [59]. In the second PCR, the diluted PCR-product (1:46) was used as a template for each bacteria specific primer pair that amplify fragments from the *Ricketsia *(27F and Rick16SR 5'-CATCCATCAGCGATAAATCTTTC-3') [60], *Spiroplasma *(27F and TKSSsp 5'-TAGCCG TGGCTTTCTGGTAA -3') [61], *Buchnera *(Buch16S1F 5'-GAGCTTGCTCTCTTTGTCGGCAA-3' and Buch16S1R 5'-CTTCTGCGGGTAACGTCACGAA-3') [60], *PASS*=*Candidatus Serratia symbiotica *(27F and PASScmp: 5'GCAATGTCTTATTAACACAT-3') [50], *Cardinium *(CLOf 5'-GCGGTGTAAAATGAGCGTG -3' and CLOr1 5'- ACCTMTTCTTAACTCAAGCCT -3' and CLOF and CLOr2 5'-TGTGTACAAGGTCCG AGAACG -3') [46], *EPS *(EPS-f 5'-TACAATCTTTATTAACCCATGTT -'3 and EPS-r 5'- TTCAAAGTAGCAAAATACATTC-3') [45] and *Wolbachia *(w76F 5'- TTGTAGCCTGCTATGGTATAACT -3' and w1012R 5' GAATAGGTATGATTTTCATGT -3') [58]. A PCR reaction for each primer pair was done according to the protocol described in the references. In each PCR plate we used sterile water as a negative control. As positive controls we used samples that were known to contain *Wolbachia *(*F. lemani*, *F. aquilonia*), but no positive control for the other bacteria was available in the beginning. In later steps samples that were shown to include the desired bacteria were used as positive control. PCR products were run on 1.5% agarose gel stained with ethidium bromide and the bands were visualized under ultraviolet illumination. The bands of a correct size were cut and purified with the MinElute Gel Extraction Kit (Qiagen). Purified DNA was cloned using TOPO TA-cloning kit (Invitrogen) and after the extraction of plasmids the success of cloning was determined by PCR [1 × buffer, 0.25 mM of each nucleotide, 0.5 uM of primer M13F and M13R and Dynazyme II (Finnzymes) 0.3 U] done in the total volume of 15 μl. The PCR program included the steps of denaturation at 94°C for 5 min, 35 cycles at 94°C for 1 min, at 46°C for 50 sec, at 72°C for 1 min and final extension at 72°C for 10 min. Three microlitres of the product was run on 1.5% agarose gel to verify the success of cloning and the correct size of the band by using M13 forward and reverse primers flanking the insertion site in the cloning vector (pCR 2.1-TOPO). The rest of the reaction was purified with the MinElute 96 UF PCR Purification Kit (Qiagen). Both strands were sequenced from PCR products from one to five clones per individual by a vector-specific primer pair (included in the TOPO TA-cloning kit) and sequencing was done with the Big Dye Ready Reaction Kit as 1/8 part reaction according to the instructions of the manufacturer (Applied Biosystems). Sequences were inspected by the program Sequencher v. 4.7 and the alignments were done in MEGA v 3.1 [54].

### Statistical analyses

The genetic relatedness (r) and inbreeding (F) coefficients for ant nests and populations were calculated using the algorithms of Queller and Goodnight [62] and Pamilo [24]. Relatedness is the probability of allele sharing between individuals above the average probability given by the gene frequencies in the population. Genetic diversity was analysed using the GENEPOP package version on the web [63] and genetic differentiation was estimated as F_ST _[64] by sampling repeatedly (20 repeats) one individual from each nest. The F_ST _estimates between populations were tested by comparing the initial estimates against the distribution obtained by permutation of the nests (100 times). The genetic distances and phylogenetic analyses were conducted using MEGA version 3.1 [54]. The haplotype network scheme for mitochondrial DNA was constructed by using the statistical parsimony method implemented in the program TCS v. 1.21. Other statistical analyses were done by using the SPSS (v 16.0) software program.

## Competing interests

The authors declare that they have no competing interests.

## Authors' contributions

AS was responsible for the laboratory work, bacteria identifications, analysis of the microsatellites, constructed the phylogenies and mt haplotype network and drafted the manuscript. PP coordinated the study, carried out the population genetic studies and helped to draft the manuscript. The final version of the manuscript was read and approved by both authors.

## Supplementary Material

Additional file 1 **UPGMA clustering of populations based on the F_ST _estimates**. Relatedness values are shown for the populations. Click here for file

Additional file 2 **Scheme of mitochondrial haplotype network for *F. cinerea *individuals**. The lines separating the haplotypes involve one nucleotide change. Click here for file

Additional file 3 **The neighbour-joining tree of *Wolbachia *16 S rDNA sequences from *F. cinerea***. The tree has sequences (896 bp) amplified from *F. cinerea *in this study and three sequences from GenBank. The tree is based on distances estimated by using the Kimura's 2-parameter method with gaps and missing data excluded from pair-wise comparisons. The numbers on the nodes represent the bootstrap percentages from 1,000 replications. Click here for file

Additional file 4 **Best matches among GenBank sequences for *Wolbachia *from *F. cinerea***. The matches are based on results from a BLAST search. Click here for file

Additional file 5 **The neighbour-joining tree of *Wolbachia *16SrDNA sequences from *Formica *ants and other arthropods**. The tree compares haplotypes obtained from *F. cinerea *and other *Formica *ants in this study and 16 sequences taken from GenBank. Click here for file

Additional file 6 **The Genbank matches for *Cardinium *sequence from *F. cinerea***. The matches are based on results from a BLAST search. Click here for file

Additional file 7 **The best matches among GenBank sequences for *Candidatus Serratia symbiotica *from *F. cinerea***. The matches are based on results from a BLAST search. Click here for file

Additional file 8 **The neighbour-joining tree of *Candidatus S. symbiotica sequences***. The tree has sequences (471 bp) amplified from *F. cinerea *in this study and one sequence from GenBank. The tree is based on distances estimated by using the Kimura's 2-parameter method with gaps and missing data excluded from pair-wise comparisons. The numbers on the nodes represent the bootstrap percentages from 1,000 replications. Click here for file
