# Supplementary figures and images for: Multiple endosymbionts in populations of the ant Formica cinerea
Source: BMC Evol Biol. 2010 Nov 1;10:335. doi: 10.1186/1471-2148-10-335 (PMC3087548; doi:10.1186/1471-2148-10-335)

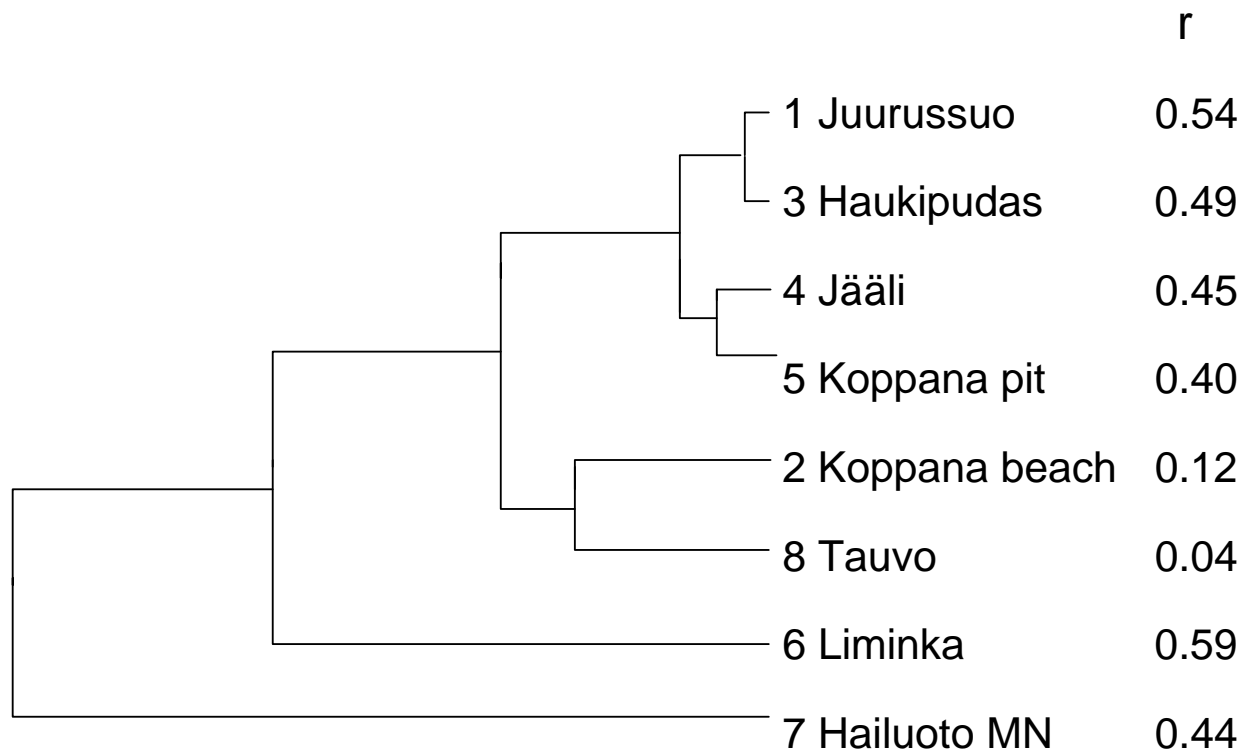

Supplement: Additional file 1 — UPGMA clustering of populations based on the FST estimates. Relatedness values are shown for the populations. [file 1471-2148-10-335-S1.PDF]

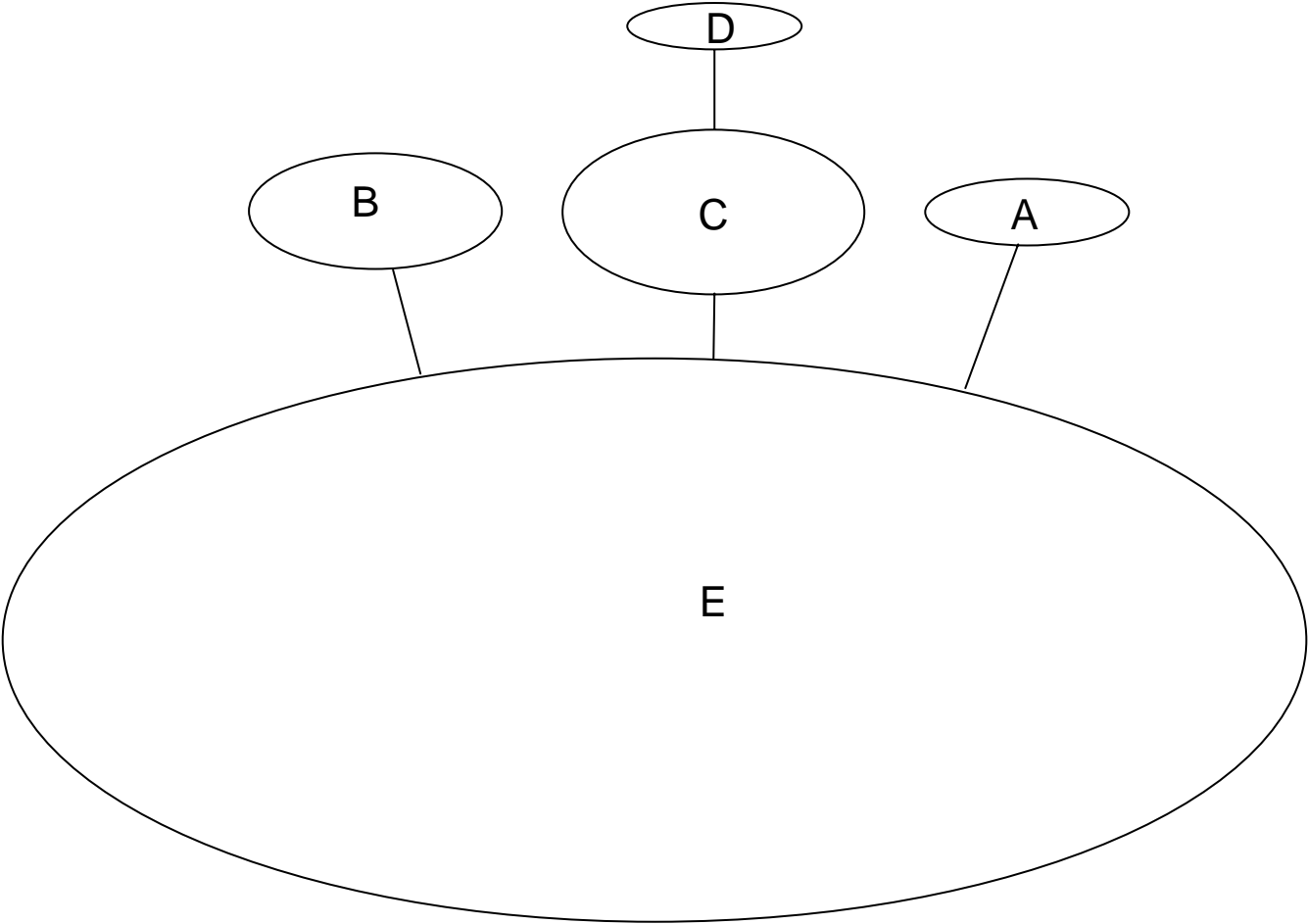

Supplement: Additional file 2 — Scheme of mitochondrial haplotype network for F. cinerea individuals. The lines separating the haplotypes involve one nucleotide change. [file 1471-2148-10-335-S2.PDF]

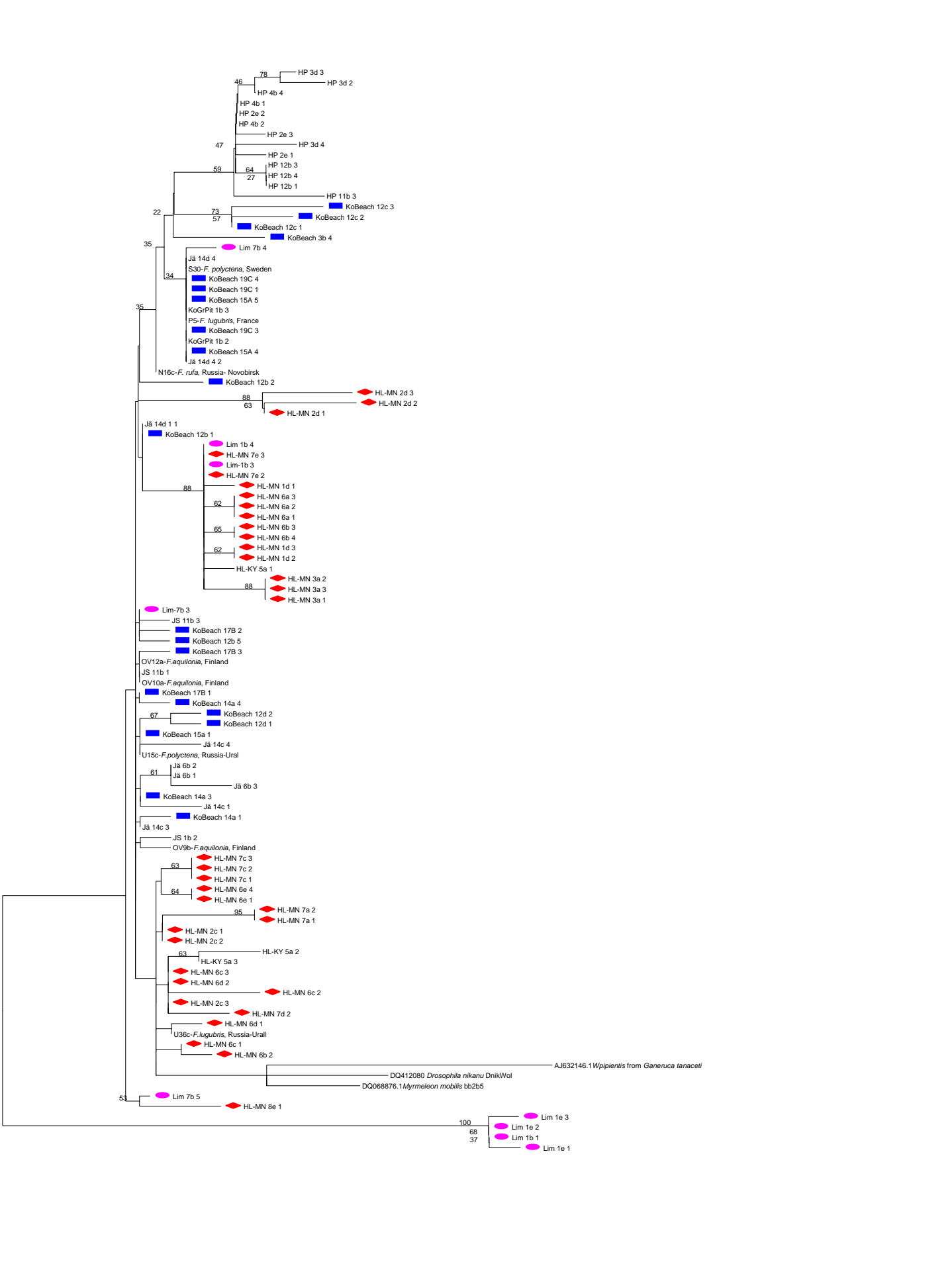

Supplement: Additional file 3 — The neighbour-joining tree of Wolbachia 16 S rDNA sequences from F. cinerea. The tree has sequences (896 bp) amplified from F. cinerea in this study and three sequences from GenBank. The tree is based on distances estimated by using the Kimura's 2-parameter method with gaps and missing data excluded from pair-wise comparisons. The numbers on the nodes represent the bootstrap percentages from 1,000 replications. [file 1471-2148-10-335-S3.PDF]

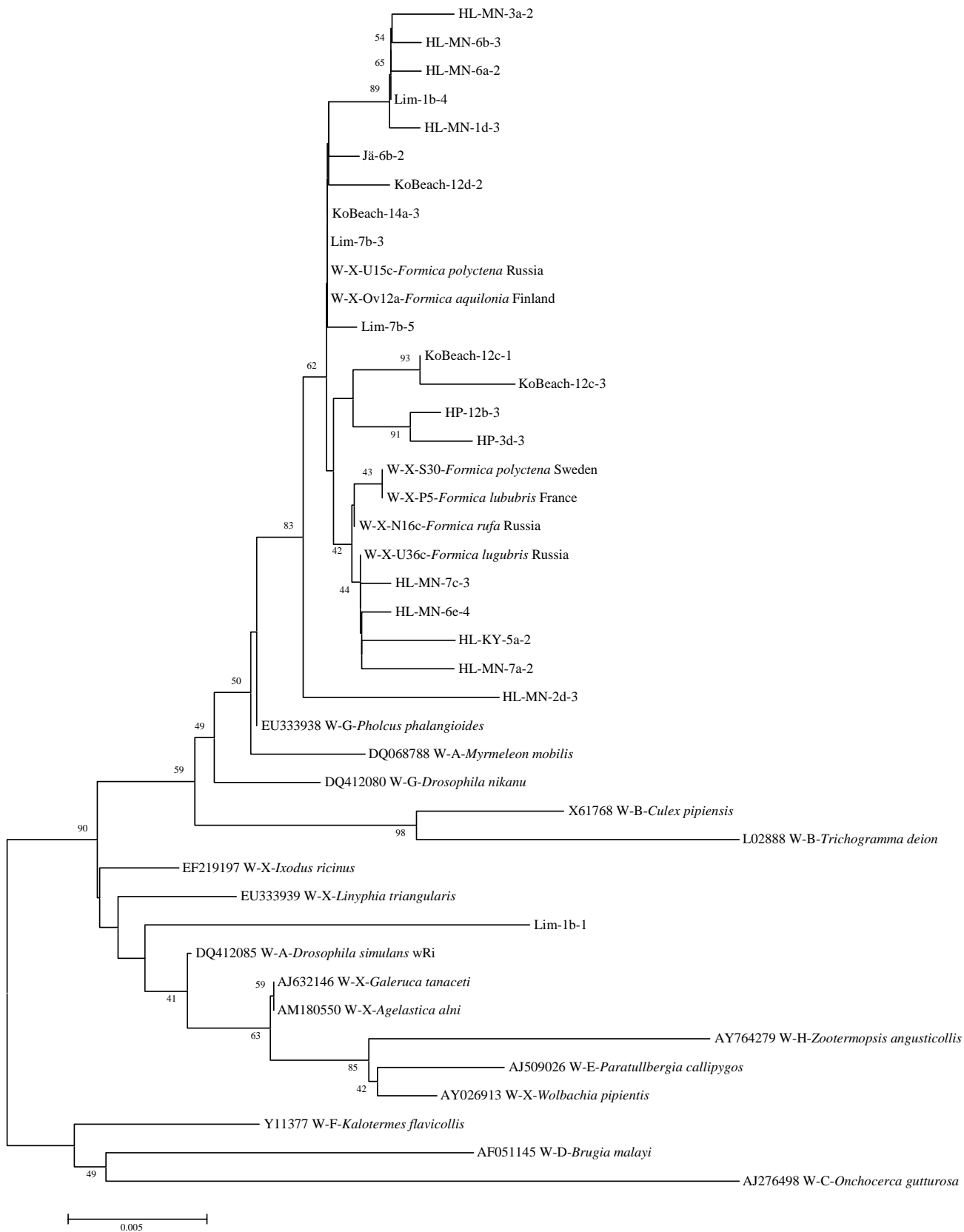

Supplement: Additional file 5 — The neighbour-joining tree of Wolbachia 16SrDNA sequences from Formica ants and other arthropods. The tree compares haplotypes obtained from F. cinerea and other Formica ants in this study and 16 sequences taken from GenBank. [file 1471-2148-10-335-S5.PDF]

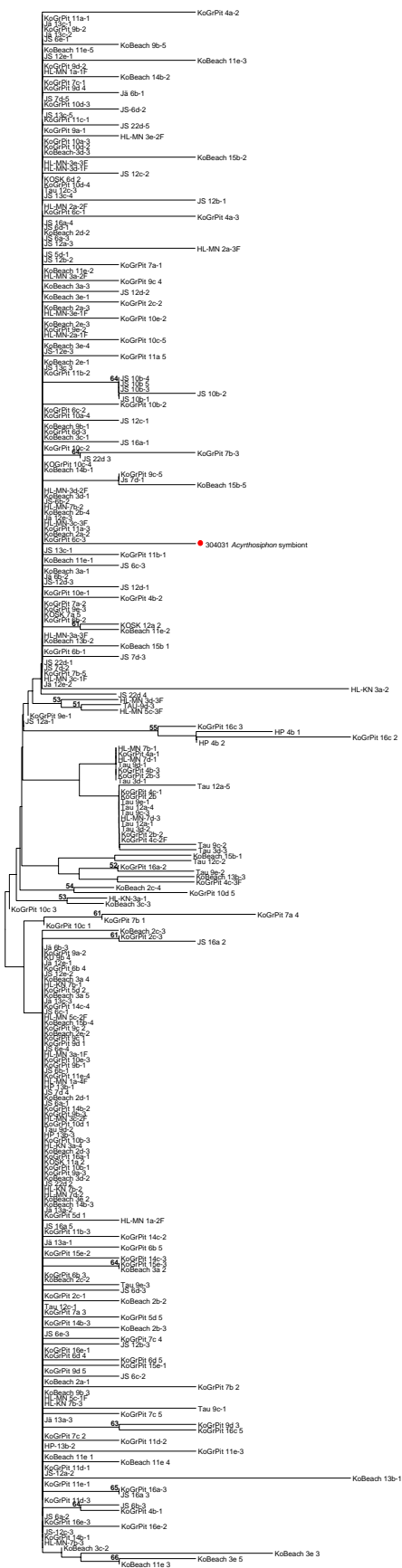

Supplement: Additional file 8 — The neighbour-joining tree of Candidatus S. symbiotica sequences. The tree has sequences (471 bp) amplified from F. cinerea in this study and one sequence from GenBank. The tree is based on distances estimated by using the Kimura's 2-parameter method with gaps and missing data excluded from pair-wise comparisons. The numbers on the nodes represent the bootstrap percentages from 1,000 replications. [file 1471-2148-10-335-S8.PDF]
