# Supplementary material for: Multiple endosymbionts in populations of the ant Formica cinerea
Source: BMC Evol Biol. 2010 Nov 1;10:335. doi: 10.1186/1471-2148-10-335 (PMC3087548; doi:10.1186/1471-2148-10-335)
Supplement: Additional file 4 — Best matches among GenBank sequences for Wolbachia from F. cinerea. The matches are based on results from a BLAST search. [file 1471-2148-10-335-S4.PDF]

Species	*Clones/strain*	*Genebank id*	***Similarity***
Cacoxenus indagator	-	EU930865.1	99%
Drosicha pinicola	DPTA3	AB491204.1	98%
Myrmeleon mobilis	bb2b5	DQ068876.1	98%
	bb2b4	DQ068875.1	
	bb2b3	DQ068874.1	
	bb2b1	DQ068873.1	
	7h4	DQ068856.1	
	1h12	DQ068823.1	
	1h1	DQ068822.1	
	f5h33	DQ068803.1	
	f4h1	DQ068790.1	
	f3h11	DQ068788.1	
	s3b7	DQ068901.1	
	f5h3	DQ068800.1	
	f6h2	DQ068812.1	
	f5h6	DQ068805.1	
	f1h9	DQ068781.1	
Drosophila nikanu	wNik	DQ412080.1	98%
D. melanogaster	wMel	DQ412083.1	98%
	trk1/dsz	AJ306310.1	
	trb1/dsz	AJ306309.1	
	hr1/dsz	AJ306308.1	
	pa1/tb	AJ306313.1	
Drosophila simulans	wRi	DQ412085.1	98%
Drosophila ananassae	wRi	DQ412084.1	98%
Drosophila	*Wolbachia pipienti*s strain EW-p	EU096232.1	98%
Diabroticite beetles	*Wolbachia* sp		
	Dcris16SWol,	AY007550.1	98%
	Ablan16SWol,	AY007548.1	
	Dlem16SWol	AY007547.1	
Ixodus ricinus	wIric 217F	EF219197.1	98%
A. diaspidis	-	X87407.1	98%
Pteromalus puparum	16SPup1	EU827689.1	98%
Nephila clavata	-	AF232234.1	98%
