# Supplementary material for: Multiple endosymbionts in populations of the ant Formica cinerea
Source: BMC Evol Biol. 2010 Nov 1;10:335. doi: 10.1186/1471-2148-10-335 (PMC3087548; doi:10.1186/1471-2148-10-335)
Supplement: Additional file 6 — The Genbank matches for Cardinium sequence from F. cinerea. The matches are based on results from a BLAST search. [file 1471-2148-10-335-S6.PDF]

Origin	*Strain/Clone*	*Genebank ID*	***Similarity***
Brevipalpus lewisi		AB116515.1	99 %
Brevipalpus californicus		AB116514	99 %
Brevipalpus phoenicis		AF350221.1	99 %
Metaseiulus occidentalis	pAJ246	AY753170.1	98 %
	pAJ238	AY753169.1	
	pAJ233	AY635291.1	
Oligonychus ilici		AB241130.1	98 %
Scaphoideus titanus		AM042540.1	98 %
Ixodes scapularis		AB001518.1	98 %
Tetranychus urticae		AB241132.1	98 %
Cardinium hertigii		DQ910766.1,	97 %
		DQ910767.1	
Tetranychus pueraricola		AB241135.1	97 %
Marietta sp		AY327470.1	97 %
