# Supplementary material for: Multiple endosymbionts in populations of the ant Formica cinerea
Source: BMC Evol Biol. 2010 Nov 1;10:335. doi: 10.1186/1471-2148-10-335 (PMC3087548; doi:10.1186/1471-2148-10-335)
Supplement: Additional file 7 — The best matches among GenBank sequences for Candidatus Serratia symbiotica from F. cinerea. The matches are based on results from a BLAST search. [file 1471-2148-10-335-S7.PDF]

Origin	*Strain/Clone*	*Genebank ID*	***Similarity***
Acyrthosiphon	symbiont S	M27040.1	98 %
Aphis craccivora	type-R	AY822593.1, AY822592.1, AY822591.1, AY822594.1	98 %
Acyrthosiphon pisum	secondary symbiont	AB033777.1, AB033778.1	98 %
Macrosiphine apdhids	*Candidatus Serratia symbiotica*	AF293617.1 AY296732.1	98 %
Uroleucon caligatum	*Candidatus Serratia symbiotica*	AF293624.1	98 %
?	*Klebsiella penumoniae* strain SWU-27	EU128493.1	97 %
Nisargruna biogas	Uncultured Pantoea sp. clone 11a	EF593045.1	97 %
Solenopsis invicta	*Serratia marcescens*	AY946291.1	97 %
Pineapple	*Klebsiella* sp.	AB114634.1	97 %
